# ER-PM Junctions on GABAergic Interneurons Are Organized by Neuregulin 2/VAP Interactions and Regulated by NMDA Receptors

**DOI:** 10.3390/ijms24032908

**Published:** 2023-02-02

**Authors:** Detlef Vullhorst, Mara S. Bloom, Neha Akella, Andres Buonanno

**Affiliations:** Section on Molecular Neurobiology, Eunice Kennedy Shriver National Institute of Child Health and Human Development, Bethesda, MD 20892, USA

**Keywords:** ER-PM junction, neuregulin 2, GABAergic interneuron, NMDA receptor, VAP, FFAT, Kv2.1

## Abstract

Neuregulins (NRGs) signal via ErbB receptors to regulate neural development, excitability, synaptic and network activity, and behaviors relevant to psychiatric disorders. Bidirectional signaling between NRG2/ErbB4 and NMDA receptors is thought to homeostatically regulate GABAergic interneurons in response to increased excitatory neurotransmission or elevated extracellular glutamate levels. Unprocessed proNRG2 forms discrete clusters on cell bodies and proximal dendrites that colocalize with the potassium channel Kv2.1 at specialized endoplasmic reticulum-plasma membrane (ER-PM) junctions, and NMDA receptor activation triggers rapid dissociation from ER-PM junctions and ectodomain shedding by ADAM10. Here, we elucidate the mechanistic basis of proNRG2 clustering at ER-PM junctions and its regulation by NMDA receptors. Importantly, we demonstrate that proNRG2 promotes the formation of ER-PM junctions by directly binding the ER-resident membrane tether VAP, like Kv2.1. The proNRG2 intracellular domain harbors two non-canonical, low-affinity sites that cooperatively mediate VAP binding. One of these is a cryptic and phosphorylation-dependent VAP binding motif that is dephosphorylated following NMDA receptor activation, thus revealing how excitatory neurotransmission promotes the dissociation of proNRG2 from ER-PM junctions. Therefore, proNRG2 and Kv2.1 can independently function as VAP-dependent organizers of neuronal ER-PM junctions. Based on these and prior studies, we propose that proNRG2 and Kv2.1 serve as co-regulated downstream effectors of NMDA receptors to homeostatically regulate GABAergic interneurons.

## 1. Introduction

Neuregulins (NRGs) are extracellular EGF-like ligands that signal via ErbB3/4 receptor tyrosine kinases to regulate numerous processes in the developing and mature nervous system including Schwann cell development and myelination [1,2,3], neurite outgrowth [4,5,6], GABAergic interneuron excitability [7,8,9,10], synaptic plasticity [11,12], visual cortex plasticity [13,14], network synchrony [15,16], and dopamine homeostasis [17,18,19], and the pathway is also genetically associated with an increased risk for psychiatric disorders [20,21]. Most NRGs are synthesized as transmembrane (TM) proforms from which the mature receptor-binding ligands are generated by proteolytic processing. Contrary to the prevailing notion that all NRGs function as axonal/presynaptic factors, we recently demonstrated that their subcellular distribution is highly distinct and determined by their transmembrane topology. Dual-pass TM isoforms such as NRG3, upon processing by BACE1, indeed traffic to glutamatergic presynaptic terminals, where they stably interact with postsynaptic ErbB4 receptors on GABAergic interneurons [22,23,24]. In stark contrast, single-pass TM NRGs such as NRG2 and NRG1 types I/II remain on the cell body and proximal dendrites as unprocessed proforms, where they cluster at endoplasmic reticulum-plasma membrane (ER-PM) junctions, an entirely unexpected finding that has been validated in vivo in various neuron types including hippocampal and cortical GABAergic interneurons, reticulothalamic neurons, and α-motorneurons [8,22,25]. Importantly, ectodomain shedding, a prerequisite for receptor binding and activation, is controlled by the excitatory neurotransmitter glutamate acting on NMDA receptors to dissociate the proform from ER-PM junctions and to stimulate the NRG sheddase ADAM10 [8,26].

ER-PM junctions are present in all cell types tested but were first identified ultrastructurally in muscle and neurons, where they are particularly abundant on cell bodies and proximal dendrites [27,28,29]. As deCamilli’s group recently reported using serial EM 3D-reconstruction, ER-PM junctions in central neurons can account for ~10% of the somatic plasma membrane surface, whereas they are far less abundant in axons and dendrites [30]. ER-PM junctions originate from protein–protein (e.g., STIM/ORAI; Kv2/VAP) and protein–lipid interactions (e.g., junctophilins, extended synaptotagmins) [31,32,33,34,35,36] and are involved in calcium homeostasis and signaling [37,38,39,40], vesicle-independent lipid transport [32,35,41], crosstalk between calcium signaling and phospholipids [42], and the regulation of muscle and neuron excitability [31,43,44]. Typically, these junctions are not associated with synapses, with the notable exception of cholinergic c-boutons on α-motoneurons, where the voltage-gated potassium channel Kv2.1, a major component of the delayed rectifier K+ current and an organizer of neuronal ER-PM junctions in mammalian neurons [45,46,47,48], functions homeostatically to control repetitive firing [49].

Our previous work showed that proNRG2 and Kv2.1 co-cluster on GABAergic interneurons [8], but the mechanisms underlying proNRG2 accumulation at ER-PM junctions and its regulation by NMDA receptors are not understood. Here, we show that proNRG2, like Kv2.1, is an organizer of ER-PM junctions by directly binding the membrane tether VAP via two sites located in its intracellular domain (ICD), one of which is phosphorylation-dependent and negatively regulated by NMDA receptor activity. Our results demonstrate that proNRG2 and Kv2.1 are coregulated, yet independent, downstream effectors of NMDA receptors and suggest that proNRG2/Kv2.1-harboring ER-PM junctions serve as a nexus for the homeostatic control of GABAergic interneuron excitability.

## 2. Results

### 2.1. ProNRG2 Organizes ER-PM Junctions in GABAergic Interneurons

**Figure 1:**
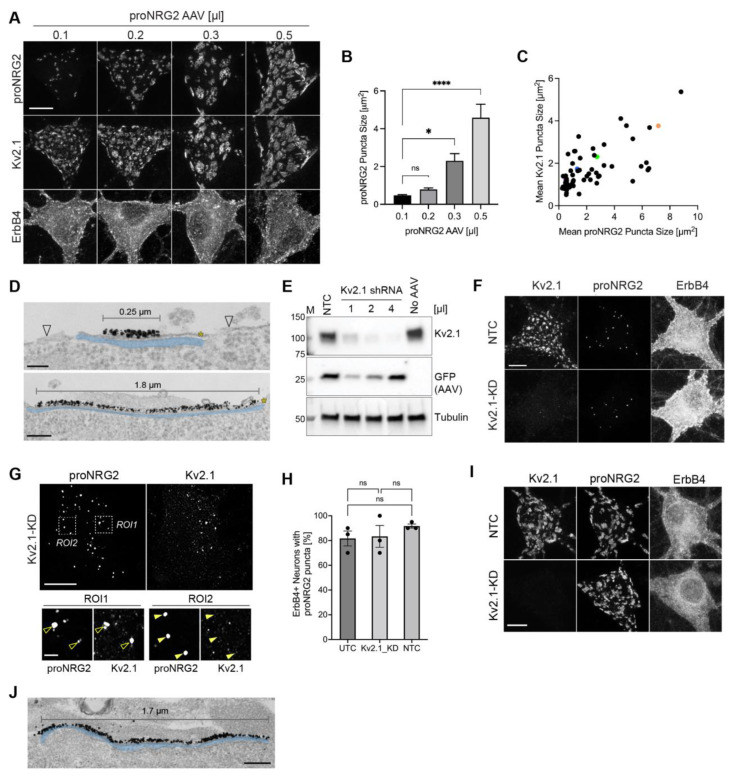
ProNRG2 organizes ER-PM junctions in ErbB4+ GABAergic interneurons. (**A**,**B**) Augmenting proNRG2 expression by AAV-mediated transduction dose-dependently increases proNRG2 cluster size without signs of diffuse surface distribution; indicated volumes are per 24-well. Note the concomitant increase in Kv2.1 cluster size that is particularly evident in the 0.5 µL condition. ProNRG2 cluster sizes shown in (**B**) represent the mean ± SEM (*n* = 713 (0.1 µL), 1211 (0.2 µL), 907 (0.3 µL), 1044 (0.5 µL) clusters from 11–13 ErbB4+ neurons per condition and two independent experiments). *, *p* < 0.05; ****, *p* < 0.0001; ns, not significant (one-way ANOVA). (**C**), Scatter plot illustrating the correlation of proNRG2 and Kv2.1 mean cluster sizes in neurons overexpressing proNRG2 (*n* = 60 neurons from three independent experiments). Colored data points are from neurons shown in panel *A* (magenta: 0.1 µL; blue: 0.2 µL; green: 0.3 µL; orange: 0.5 µL AAV). (**D**), Representative electron micrographs showing dense patches of the proNRG2 immunogold signal at the plasma membrane (marked by asterisks) that align with the underlying SSC. The top micrograph shows a small patch of proNRG2 signal that is concentrated over the center of the SSC while sparing its periphery. This distribution resembles endogenous proNRG2 that is frequently found at the open center of doughnut-shaped Kv2.1 clusters [8]. Note the lack of signal outside of the junctional region (arrowheads) and the extensive alignment of the very large proNRG2 cluster with the underlying SSC in the bottom image. See also Figure S1 for uncropped overviews and magnified areas without SSC shading. (**E**), shRNA-mediated knockdown dramatically reduces Kv2.1 protein levels in AAV-transduced neurons expressing a potent Kv2.1 shRNA compared to neurons transduced with nontargeting control (NTC) or untransduced neurons (UTC). GFP and tubulin signals included as transduction and loading controls, respectively. (**F**), Similar proNRG2 clusters are found in ErbB4+ GABAergic interneurons under both control (NTC) and Kv2.1 knockdown conditions (Kv2.1-KD). (**G**), Superresolution (Airyscan) micrograph of a Kv2.1 knockdown neuron, with two regions of interest magnified below, showing examples of proNRG2 puncta either accompanied by a residual Kv2.1 signal (ROI1; open arrowheads) or lacking a detectable Kv2.1 signal (ROI2; filled arrowheads). (**H**), Kv2.1 knockdown does not affect the number of ErbB4+ neurons with proNRG2 puncta compared to nontargeting (UTC) or untransduced control (NTC) neurons; data represent the mean ± SEM of 275 (NTC), 250 (Kv2.1-KD), and 245 (UTC) neurons from three independent experiments. ns, *p* > 0.05 (Kruskal–Wallis test). (**I**), Kv2.1 knockdown does not affect the clustering of overexpressed proNRG2 in neurons co-transduced with AAVs expressing shRNA and proNRG2. (**J**), Electron micrograph of a Kv2.1 knockdown neuron showing an extensive patch of proNRG2 immunogold signal at the plasma membrane that is accompanied by a similarly long SSC. See also Figure S1 for uncropped overviews and magnified areas without SSC shading. Scale bars: (**A**,**F**,**G**) (overviews) and (**I**) = 10 µm; (**D**) = 100 nm (top) and 200 nm (bottom); (**G**) = 2 µm (ROIs); (**J**) = 200 nm.

Cultured neurons are a biologically relevant and experimentally accessible model system for exploring the mechanisms underlying proNRG2 accumulation at neuronal ER-PM junctions. Because endogenous NRG2 is co-expressed along with its receptor ErbB4, which, in cultures, is exclusively expressed in GABAergic interneurons [8], we focused our analyses on ErbB4+ neurons. We began by testing whether proNRG2 overexpression by AAV-mediated transduction promotes the formation of junctions in cultured hippocampal neurons (see Section 4 for information regarding AAV titrations). As shown in Figure 1A,B, using a range of AAV transduction volumes (0.1–0.5 µL), we found proNRG2 clusters on the cell body and proximal dendrites that dose-dependently increased in size from a mean of 0.47 ± 0.04 µm^2^ (SEM) at 0.1 µL to 4.58 ± 0.72 µm^2^ at 0.5 µL, without signs of diffuse distribution (see also Figure S3 (top) for a low-magnification micrograph of an ErbB4+ neuron overexpressing proNRG2, illustrating the complete absence of clusters in more distal dendrites). Unexpectedly, this effect was accompanied by a similar increase in the Kv2.1 cluster size (Figure 1C; Pearson correlation coefficient = 0.67; *p* < 0.0001). To test whether proNRG2 clusters localize to bona fide ER-PM junctions, we investigated their distribution on the plasma membrane by surface-labeling the NRG2 extracellular domain with a monoclonal antibody [8] and analyzed the signals by immunogold electron microscopy. Indeed, as shown in Figure 1D and Figure S1, proNRG2 immunogold signals were detected on the plasma membrane, where they were confined to densely labeled patches of various lengths that were apposed by flattened single stacks of ER, also known as subsurface cisterns or SSCs, that closely align with the plasma membrane. This organization is characteristic of ER-PM junctions.

Clustered Kv2.1 has recently been reported to act as an organizer of neuronal ER-PM junctions [46,47,48]. We therefore asked whether Kv2.1 contributes to proNRG2 accumulation at ER-PM junctions using shRNA-mediated gene knockdown. Toward this end, we developed an AAV harboring a potent shRNA targeting the 3′-untranslated region of rat Kv2.1 (Kv2.1_2956; see Section 4) and confirmed by Western blotting that Kv2.1 protein was dramatically reduced to 20 ± 8% (SEM; *n* = three independent experiments) of control levels in knockdown neurons transduced with 4 µL Kv2.1_2956 AAV per 24-well compared to untransduced neurons (Figure 1E). We then analyzed the subcellular distribution of endogenous proNRG2 in AAV-transduced ErbB4+ GABAergic interneurons by conventional confocal (Figure 1F) and Airyscan superresolution microscopy (Figure 1G). We found that Kv2.1 knockdown did not affect the ability of proNRG2 to form highly discrete puncta on the cell body (Figure 1G,H), although quantitative analyses indicated that the number of proNRG2 puncta per neuron and the mean puncta size were moderately reduced (Figure S2; see also Discussion). Likewise, the ability of AAV-overexpressed proNRG2 to form large clusters was similarly unaffected by Kv2.1 knockdown (Figure 1I), and immunogold electron microscopy confirmed that proNRG2 clusters in Kv2.1 knockdown neurons were accompanied by SSCs and therefore represent bona fide ER-PM junctions (Figure 1J and Figure S1). Together, these results demonstrate that proNRG2, like Kv2.1, can organize ER-PM junctions in GABAergic interneurons.

### 2.2. VAP Co-Immunoprecipitates with proNRG2

**Figure 2:**
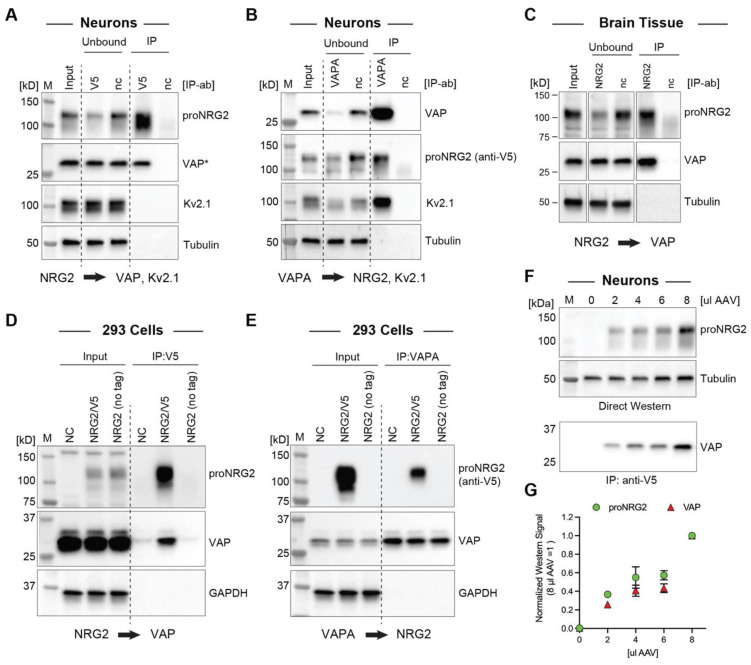
ProNRG2 and VAP co-immunoprecipitate in cultured neurons, brain tissue, and heterologous cells. (**A**), IP of epitope-tagged proNRG2 from transduced neuron lysates co-immunoprecipitates VAP detected with mouse monoclonal antibody clone N479/107 against VAPA/B [48], whereas VAP is not detected in the negative control IP lane (*nc*) using an isotype-matched negative control antibody (anti-GFP; clone N86/6). Importantly, unlike VAP, Kv2.1 is not detected in the V5-IP lane. The asterisk (*) indicates that input and unbound fractions were diluted fivefold for VAP detection. (**B**), Likewise, proNRG2 is detected along with Kv2.1 in parallel VAPA-IP samples prepared from the same neuron lysates. (**C**), VAP co-immunoprecipitates with proNRG2 from mouse whole brain lysates following IP with a rabbit polyclonal NRG2 antibody against the extracellular domain (ABN1654). *nc*, negative control antibody (normal rabbit IgG). (**D**,**E**), ProNRG2 and VAP also co-immunoprecipitate in NRG2-transfected 293 cells. IP controls include lysates prepared from cells transfected with proNRG2 lacking the V5 epitope used for IP (no tag) and negative control IP antibodies (anti-GFP in (**D**); normal rabbit IgG in (**E**)). (**F**), Increasing proNRG2 expression in AAV-transduced neurons (0–8 µL per six-well) dose-dependently augments VAP co-immunoprecipitation signals. (**G**), Densitometric analysis of proNRG2/VAP signals shown in (**F**). Data are normalized to values obtained for 8 µL AAV (*n* = three independent experiments).

The above findings strongly suggested that proNRG2 directly interacts with an ER-resident protein tether. Considering the extensive colocalization of Kv2.1 with proNRG2 (this work; [8,26]) and the interaction of Kv2.1 with VAP at neuronal ER-PM junctions [47,48], we explored if proNRG2 directly binds VAP using a co-immunoprecipitation approach. Because endogenous proNRG2 protein levels in cultured hippocampal neurons are too low for Western blotting detection, we boosted the expression by AAV-mediated transduction of V5 epitope-tagged proNRG2. The validity of this approach was supported by our earlier immunofluorescence and electron microscopy data showing that proNRG2 is confined to ER-PM junctions, even when overexpressed (see Figure 1). Indeed, proNRG2 IP with anti-V5 successfully co-immunoprecipitated VAP, and, conversely, VAP IP with an anti-VAPA antibody co-immunoprecipitated proNRG2 (Figure 2A,B). Importantly, Kv2.1 was completely absent from proNRG2-IPs (Figure 2A), whereas it was readily detected in VAP-IPs (Figure 2B), suggesting that proNRG2 does not directly interact with Kv2.1. To test whether endogenous proNRG2 and VAP interact in vivo, we performed IP experiments in mouse whole brain lysates using a NRG2 extracellular domain antibody [8]. As shown in Figure 2C, VAP was detected in anti-NRG2 but not in negative control antibody immunoprecipitates, demonstrating that both proteins interact endogenously in brain tissue. Furthermore, proNRG2 and VAP also co-immunoprecipitated in transfected 293 cells that lack Kv2.1 (Figure 2D,E). Together, these findings strongly suggest that VAP directly tethers proNRG2 to ER-PM junctions.

Our immunocytochemistry data (see Figure 1A,B) demonstrated that overexpression dose-dependently increases the proNRG2 cluster size without any indication of diffuse surface distribution. We therefore reasoned that proNRG2 cluster size reflects the extent of VAP recruitment and tested this idea by transducing neurons with different amounts of proNRG2 AAV. As shown in Figure 2F,G, proNRG2 signals in cell lysates indeed correlate well with VAP signals in the corresponding co-immunoprecipitation samples, indicating that VAP is sufficiently abundant to support the formation of ER-PM junctions, even under conditions of strongly increased proNRG2 expression.

### 2.3. ProNRG2 Binds Both VAPA and VAPB via FFAT-Type Interactions

**Figure 3:**
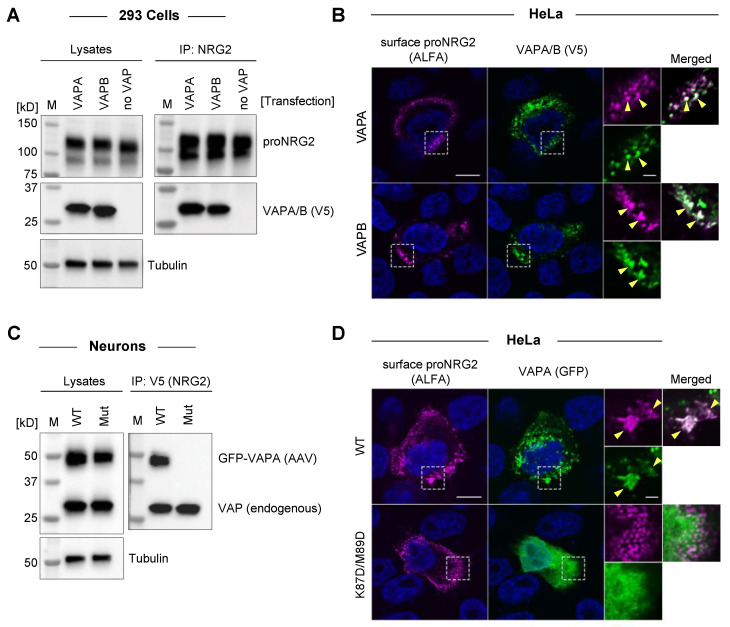
ProNRG2 interacts with VAPA and VAPB isoforms, and binding requires an intact FFAT binding site in VAP. (**A**), ProNRG2 co-immunoprecipitates both VAPA and VAPB in 293 cells transfected with untagged proNRG2 and V5-tagged VAPA/B, as indicated. Cells in the negative control lanes were transfected with proNRG2 but not VAP (no VAP). (**B**), Single-plane confocal micrographs showing colocalization of VAPA clusters (top) and VAPB clusters (bottom) with surface proNRG2 in transfected HeLa cells. Arrowheads in magnified ROIs indicate examples of overlapping proNRG2/VAP signals. Note that proNRG2 was live-labeled with a fluorescently conjugated nanobody against the ALFA tag inserted upstream of the EGF-like domain. DAPI was included to label nuclei. (**C**), ProNRG2/VAP co-immunoprecipitation requires a functional FFAT binding site. Neurons were transduced with AAVs for V5-tagged proNRG2 and GFP-tagged wild-type (WT) or mutant (Mut; K87D/M89D) VAPA. Note that endogenous VAP is detected in both IP samples. (**D**), Single-plane confocal micrographs showing co-clustering of WT VAPA-GFP with ALFA-tagged proNRG2 (top; arrows in ROI) but not of mutant VAPA-GFP (bottom) in transfected HeLa cells. ProNRG2 was live-labeled as described in panel (**B**), and DAPI was included to label nuclei. Magnified ROIs in (**B**,**D**) include single and merged channels for proNRG2/alfa and VAP-GFP. Scale bars in (**B**,**D**): overviews = 10 µm; ROIs = 2 µm.

Mammalian cells express three different VAP isoforms. VAPA and VAPB are highly homologous ER transmembrane proteins, whereas VAPC is a splice variant of VAPB that lacks the TM domain and is therefore unable to tether membranes [50]. To test whether proNRG2 interacts with both VAPA and VAPB isoforms, we performed co-immunoprecipitation experiments using anti-NRG2 (see Figure 2) and lysates from 293 cells co-transfected with untagged proNRG2 and V5-tagged VAPA or VAPB. As shown in Figure 3A, proNRG2 co-immunoprecipitated both VAPA and VAPB with a similar efficiency, as judged by the comparable VAPA and VAPB band intensities between whole-cell lysates and IP lanes.

Furthermore, both VAPA and VAPB isoforms co-clustered with surface-labeled proNRG2 at presumed ER-PM junctions in transfected HeLa cells (Figure 3B).

Canonical VAP interactions involve an electro-positive area within the MSP domain that interacts with FFAT and FFAT-like motifs [51]. To test whether proNRG2–VAP interactions require a functional FFAT binding site, we transduced neurons with V5-tagged proNRG2 and GFP-tagged wild-type (WT) or mutant VAPA harboring two point mutations in the MSP domain (K87D/M89D; [52]). As shown in Figure 3C, anti-V5 co-immunoprecipitated both endogenous VAP and WT but not mutant GFP-VAPA. Likewise, only WT VAPA-GFP, but not its mutated variant, co-clustered with surface-labeled proNRG2 in transfected HeLa cells (Figure 3D).

### 2.4. Both C- and D-Boxes in the ICD Are Necessary for High-Affinity Interactions of proNRG2 with VAP

**Figure 4:**
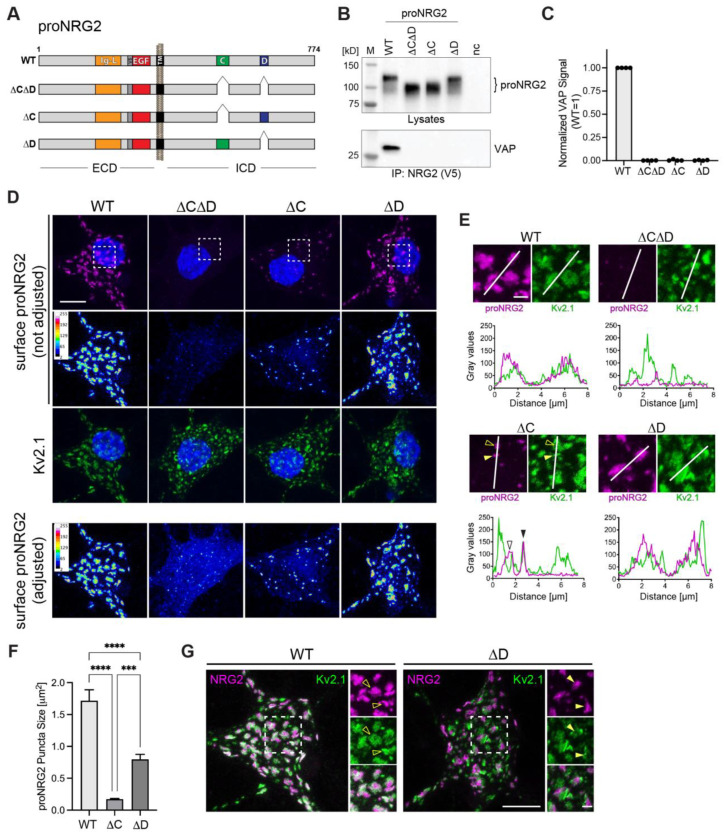
Role of C- and D-boxes in proNRG2/VAP interactions. (**A**), Schematic illustration of proNRG2 and C/D-box deletions constructs. Locations of C- and D-boxes in the ICD, EGF-like (EGF) and Ig-like (Ig-L) domains in the ECD, transmembrane domain (TM), and V5 epitope tag used for IP are shown. (**B**), Representative Western blot confirming comparable expression levels of proNRG2 constructs in neuron lysates (top) and showing corresponding VAP co-immunoprecipitation results (bottom). *nc*, untransduced control. (**C**), Densitometric quantification of VAP co-immunoprecipitation band intensities; note that only WT proNRG2 co-immunoprecipitates VAP. Data are normalized to VAP co-immunoprecipitation signals obtained with WT proNRG2 (*n* = four independent experiments). (**D**), Confocal micrographs of surface-labeled proNRG2 showing differences in clustering between WT and deletion variants. ProNRG2 signals in the panels above were obtained using the same laser intensity across all variants and are shown in magenta (top) and as a 16-color heat map (middle). Kv2.1 signals in green are included for reference (bottom). The second heat map in the panel below was obtained by individually adjusting laser intensities for each proNRG2 variant to better visualize diffusely distributed low-intensity signals obtained with the ΔCΔD and, to a lesser extent, the ΔC variants. ROIs used for line scan densitometry in panel (**E**) are indicated. (**E**), Magnified ROIs from panel (**D**), showing the location of the lines used for densitometry (top) and the corresponding line scan densitometry graphs (bottom). Filled and open arrowheads in the ΔC panel indicate examples of proNRG2 clusters that respectively colocalize with Kv2.1 or that are located in the open centers of doughnut-shaped Kv2.1 clusters. By contrast, theΔD variant readily clusters, although clusters co-localize only partially with Kv2.1 (see also panel (**G**). (**F**), Quantitative analysis of proNRG2 cluster size. Data represent the means ± SEM (# of clusters analyzed: 2197 (WT); 798 (ΔC); 2726 (ΔD)) from 23–24 ErbB4+ neurons per variant and two independent experiments. The ΔCΔD variant was not included, as most analyzed neurons lacked detectable clusters. ***, *p* < 0.001; ****, *p* < 0.0001 (one-way ANOVA). (**G**), Unlike WT proNRG2, which overlaps extensively with Kv2.1 (left, open arrowheads), clustered proNRG2ΔD signals are strongest in areas immediately adjacent to Kv2.1 clusters (right, filled arrowheads). ROIs indicated in overview images are magnified on the right and shown as separate and merged channels. Scale bars: 10 µm ((**D**), overviews in (**G**)) and 2 µm ((**E**), ROIs in (**G**)).

The ICDs of proNRG2 and many NRG1 isoforms harbor two highly conserved motifs denoted as C- and D-boxes [53], and the deletion of an area in the ICD that includes these sequences abolishes proNRG2 accumulation at ER-PM junctions [26]. To directly test their involvement in VAP binding, we generated AAVs harboring three deletion variants that selectively target the C- and D-boxes, either in combination (proNRG2ΔCΔD) or individually (proNRG2ΔC and proNRG2ΔD) (Figure 4A). Co-immunoprecipitation experiments were performed on lysates from transduced neurons using anti-V5, as described above. Although all proNRG2 variants were expressed at similar levels, only WT proNRG2, but none of the deletion variants, was able to co-immunoprecipitate VAP, strongly suggesting that both the C- and D-boxes are required for VAP binding (Figure 4B,C). We then investigated the subcellular distribution of these proNRG2 deletion variants by confocal microscopy to determine the extent to which the C- and D-boxes are necessary for clustering at ER-PM junctions. Transduced neurons were first surface-labeled for proNRG2 and then labeled for Kv2.1 following permeabilization to identify ER-PM junctions. When using imaging conditions that yield minimal saturation for WT proNRG2 clusters (Figure 4D; top), signals for the surface proNRG2ΔCΔD were very low. However, when laser power (Figure 4D; bottom) or expression levels were increased (Figure S3), surface proNRG2ΔCΔD was more readily observed and was found to broadly distribute throughout the soma and dendrites without any apparent accumulation at ER-PM junctions. By contrast, proNRG2ΔC formed small puncta that colocalized with larger Kv2.1 clusters (ΔC: 0.17 ± 0.01 µm^2^ vs. WT: 1.71 ± 0.17 µm^2^; Figure 4D–F). Unexpectedly, clustering of proNRG2ΔD was only moderately reduced (ΔD: 0.80 ± 0.08 µm^2;^ Figure 4D–F). However, unlike WT proNRG2, these clusters frequently resided in areas with relatively weak Kv2.1 immunoreactivity that were adjacent to stronger Kv2.1 signals (Figure 4E,G), suggesting that proNRG2ΔD and Kv2.1 localize to the same ER-PM junctions but preferentially accumulate in distinct microdomains. Taken together with our co-immunoprecipitation data, these findings indicate that, in situ, the C-box plays a major role and the D-box plays a minor role in mediating proNRG2-VAP interactions, but cooperative binding via both sequence elements is necessary to form proNRG2/VAP complexes that persist under the more stringent conditions of immunoprecipitation (i.e., in the presence of 1% Triton X-100 and at low protein concentrations in diluted cell lysates).

### 2.5. The C-Box Contains a Cryptic and Phosphorylation-Dependent VAP Binding Site

**Figure 5:**
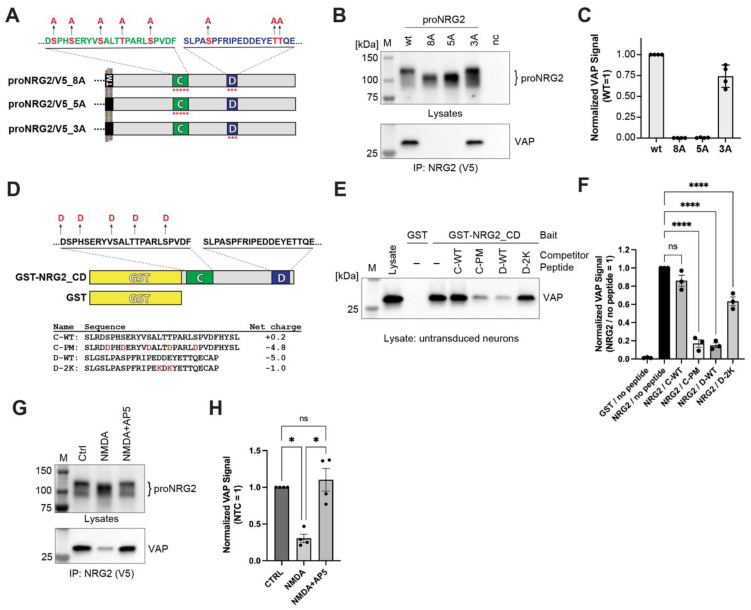
The proNRG2 C-box harbors a phosphorylation-dependent VAP binding site, and VAP binding is regulated by NMDA receptor activity. (**A**), Schematic illustration of WT and point-mutated C- and D-box sequences in the proNRG2 ICD, with targeted putative Ser/Thr phosphorylation sites indicated. (**B**), Representative co-immunoprecipitation Western blot of AAV-transduced neurons showing proNRG2 signals in cell lysates above and VAP co-immunoprecipitation signals below. The blot illustrates how rendering Ser/Thr residues in the C-box (proNRG2_8A and 5A), but not the D-box (proNRG2_3A), nonphosphorylatable affects proNRG2 electrophoretic mobility and abolishes VAP co-immunoprecipitation. (**C**), Densitometric analysis of VAP co-immunoprecipitation signals. Data are normalized to WT proNRG2 and represent the mean ± SD (*n* = four independent experiments). (**D**), Schematic illustration of GST-NRG2_CD and GST-only negative control bait proteins used in pulldown experiments. Ser/Thr -> Asp substitutions in the C-box are indicated above, and the sequences as well as the net charges of competing C- and D-box peptides are shown below (mutated residues highlighted in red). (**E**), Representative Western blot showing VAP signals after pulldown from untransduced neuron lysates with GST or GST-NRG2_CD bait proteins, either in the absence or presence of competing peptides, as indicated. Note that only the phospho-mimicking C-box peptide C-PM and the WT D-box peptide successfully compete for VAP binding, whereas neither C-WT nor D-2K peptides compete. (**F**), Summary analysis of representative results shown in (**E**). Data are plotted as normalized VAP signal intensities (NRG2/no peptide = 1) and represent the mean ± SEM (*n* = three independent experiments). (**G**), NMDA receptor stimulation (NMDA; 50 µM) increases crNRG2 electrophoretic mobility indicative of dephosphorylation (top) and decreases VAP signals in the corresponding immunoprecipitation samples (bottom). (**H**), Summary analysis of representative results shown in (**G**). Data are plotted as normalized VAP signal intensities (Ctrl = 1) and represent the mean ± SEM (*n* = four independent experiments). *, *p* < 0.05; ****, *p* < 0.0001 (one-way ANOVA).

Inspection of the C- and D-box sequences failed to reveal overt similarities with canonical FFAT motifs (“two phenylalanines in an acidic tract”, EFFDAxE; [51]). However, the D-box harbors a tract of acidic residues (EDDEYE) that imparts a strongly negative net charge (−5.0) and therefore conceivably represents a noncanonical FFAT-like motif (Figure 5A). By contrast, the C-box lacks an acidic tract and has a near-neutral net charge of +0.2. We previously reported that NMDA receptor activation promotes the dephosphorylation of Ser/Thr residues in the C- and/or D-boxes, thereby dissociating proNRG2 from ER-PM junctions [26], and Kv2.1-VAP interactions have been shown to require the phosphorylation of Ser residues in its proximal restriction and clustering (PRC) domain to convert a cryptic site into a functional VAP binding site [47,48]. We therefore set out to test the role of phosphorylation in proNRG2/VAP interactions by changing all eight potential Ser/Thr phosphorylation sites in the C- and D-boxes (identified with the NetPhos-3.1 phosphorylation site prediction tool) to Ala to render them non-phosphorylatable (proNRG2_8A; Figure 5A). Consistent with our earlier findings [26], we found that proNRG2_8A shows a noticeable increase in SDS-PAGE electrophoretic mobility compared to WT proNRG2 despite minimal differences in their respective molecular masses (Figure 5B). As hyperphosphorylated proteins migrate with reduced mobility [54], this change in electrophoretic mobility is indicative of a loss of negative charge and is consistent with the notion that at least some of the targeted Ser/Thr residues are phosphorylated. Strikingly, this mobility change was accompanied by a complete loss of VAP signal in the corresponding IP lane, indicating that phosphorylation is required for stable proNRG2/VAP interactions (Figure 5B,C). Next, we selectively targeted Ser/Thr residues in the C-box (proNRG2_5A; Figure 5A). Like proNRG2_8A, the electrophoretic mobility of proNRG2_5A was increased relative to WT proNRG2, and VAP was absent from the corresponding IP reaction (Figure 5B,C). By contrast, the proNRG2_3A variant harboring Ser/Thr->Ala mutations selectively in the D-box lacked the electrophoretic mobility shift and largely retained the ability to co-immunoprecipitate VAP (Figure 5B,C). Together, these data strongly suggest that Ser/Thr residues in the C-box, but not in the D-box, are phosphorylated and that phosphorylation is required to turn a cryptic site into a functional VAP binding motif.

Next, we used GST pulldowns, combined with peptide competition, to further investigate the role of negative charges in proNRG2/VAP interactions. We began by generating a GST fusion protein that encompasses the entire sequence between the C- and the D-box, and in which the aforementioned Ser/Thr residues in the C-box were replaced by Asp to render them negatively charged (Figure 5D; see Section 4 for details). Unlike the unfused GST negative control, this bait protein was able to pull down VAP from lysates of untransduced neurons in the absence of any competing peptides (Figure 5E,F). A peptide encompassing the WT C-box sequence (C-WT; Figure 5D) was unable to compete for binding to VAP, as evidenced by similar VAP signal intensities in the no-peptide and C-WT lanes. By contrast, the corresponding phospho-mimicking peptide C-PM was effective in competing with VAP for binding to the bait protein, demonstrating that the addition of a negative charge is essential to rendering the C-box VAP binding competent. Conversely, the WT D-box peptide (D-WT) effectively competed with the bait protein for VAP binding (Figure 5E,F). However, a D-box peptide whose net charge was reduced from −5.0 to −1.0 by converting two acidic amino acids (D^620^ and E^622^) into lysines (D-2K; Figure 5D) was a much weaker competitor (Figure 5E,F). Taken together with the point mutations experiments described above, these competition experiments provide further evidence that the VAP binding motif in the C-box requires phosphorylation to efficiently interact with VAP.

### 2.6. NMDA Receptors Regulate the Association of proNRG2 with VAP

In neurons, signaling processes downstream of NMDA receptor activation mediate Ser/Thr dephosphorylation of proNRG2 and its dissociation from ER-PM junctions [26]. We therefore asked whether acute treatment with the agonist NMDA (50 µM) promotes the dissociation of proNRG2/VAP complexes in transduced neurons. For these experiments, we used a cleavage-resistant variant of proNRG2 (crNRG2) to circumvent NMDA receptor-dependent NRG2 ectodomain processing by ADAM10 [26]. As shown in Figure 5G,H, 10 min of NMDA application caused a noticeable downward shift of the crNRG2 band, indicative of dephosphorylation. Importantly, this treatment also significantly reduced normalized VAP signals in the corresponding co-immunoprecipitation experiments to 0.31 ± 0.06 (SEM). This effect was blocked by the selective NMDA receptor antagonist D-AP5 (50 µM; 1.10 ± 0.31). Together, these findings strongly suggest that NMDA receptor activity dissociates proNRG2 from ER-PM membrane junctions by disrupting its interaction with VAP through dephosphorylation of Ser/Thr residues in its C-box.

## 3. Discussion

In this study we demonstrate that proNRG2 functions as an activity-dependent organizer of ER-PM junctions in GABAergic interneurons by directly interacting with the membrane tether VAP. While based primarily on experiments performed in cultured neurons and cell lines, our previous studies showing proNRG2 accumulation at ER-PM junctions in various neuron types in the CNS [8,22], together with the demonstration of proNRG2-VAP complexes in whole brain protein extracts (Figure 2), suggest that these findings are pertinent to proNRG2 biology in the intact brain. Our work thus adds proNRG2 and potentially proforms of other single-pass TM NRGs to a growing list of neuronal membrane tethers. The interaction between proNRG2 and VAP is unconventional, combining two low-affinity sites and posttranslational modification to render its residence at ER-PM junctions highly sensitive to changes in NMDA receptor activity. As discussed further below, our results suggest that proNRG2 and Kv2.1 act cooperatively in GABAergic interneurons to homeostatically tune their activity in response to changes in excitatory activity and extracellular glutamate levels.

### 3.1. ProNRG2 Is an Activity-Dependent Organizer of Neuronal ER-PM Junctions

Until recently, all neuronal NRG isoforms were thought to act as presynaptic or axonal signaling factors by signaling via postsynaptic ErbB4 or glial ErbB2/3 receptors [55]. Our earlier studies on CNS neurons [8,26] and studies on α-motoneuron C-boutons [22,25] recently challenged this dogma by demonstrating that proNRG2 and single-pass TM NRG1 isoforms do not traffic to axons but rather accumulate at neuronal ER-PM junctions on cell bodies and proximal dendrites. Although these junctions arise from a multitude of protein–protein and protein–lipid interactions [32,35], the colocalization and NMDA receptor regulation of proNRG2 and Kv2.1 suggested that proNRG2 either requires Kv2.1 for accumulation at junctions or that both proteins share the same tethering mechanism. Several lines of evidence from this study support the latter idea, including: (1) proNRG2 overexpression promotes the formation of ER-PM junctions in cultured neurons; (2) shRNA-mediated Kv2.1 knockdown does not result in proNRG2 declustering; (3) proNRG2 co-immunoprecipitates VAP but not Kv2.1; and (4) proNRG2 interacts and colocalizes with VAP in heterologous 293 and HeLa cell lines that lack Kv2.1. Interestingly, we observed a moderate reduction in proNRG2 puncta in Kv2.1 knockdown neurons (see Figure S2). This is likely a secondary effect resulting from increased neuron excitability following Kv2.1 knockdown/knockout [56,57], which would augment the activity-dependent dissociation of proNRG2 from ER-PM junctions and its cleavage by ADAM10 [8,26].

Together, our molecular and cell biological data firmly establish that proNRG2 and Kv2.1 cooperatively organize ER-PM junctions in GABAergic interneurons. Whether the NMDA receptor-mediated disruption of proNRG2-VAP interactions merely triggers their dissociation from these junctions or causes the junctional membranes to separate was not directly tested in this study. However, the available evidence from light and electron microscopy imaging studies strongly supports the latter [46,48,58]. Lastly, while other VAP-interacting proteins and L-type Ca^2+^ channels have been reported to accumulate at Kv2.1+ ER-PM junctions [39,59,60], it will be interesting in future studies to investigate to what extent these VAP-based junctions are distinct from junctions mediated by other membrane tethers and, more broadly, whether different membrane tethers define spatially and functionally distinct subpopulations of ER-PM junctions.

### 3.2. Features of proNRG2/VAP Interaction and Its Regulation by NMDA Receptors

We previously delineated a 138 amino acid region in the proNRG2 ICD as critical in restricting subcellular proNRG2 distribution to ER-PM junctions [26]. This region encompasses the C- and D-boxes that are highly conserved between NRG1/2 orthologs and across species [53]. By selectively deleting them, we demonstrated here that the C- and D-boxes cooperatively mediate proNRG2 clustering at ER-PM junctions. Whereas both motifs are critical for stable VAP interactions in vitro (i.e., under co-immunoprecipitation conditions), we unexpectedly found that either motif is sufficient to support proNRG2 clustering at ER-PM junctions in situ (i.e., under immunocytochemistry conditions), albeit at reduced levels. These experiments also revealed that the deletion of the C-box is more detrimental for clustering than the deletion of the D-box, indicating that the C-box plays a predominant role in VAP binding (Figure 4). To what extent the apparent lower levels of surface proNRG2 observed with the ΔCΔD and ΔC deletion variants are due to deficient clustering, reduced surface trafficking and/or retention, or increased ectodomain shedding is an interesting question but one that is outside the scope of this study.

**Figure 6:**
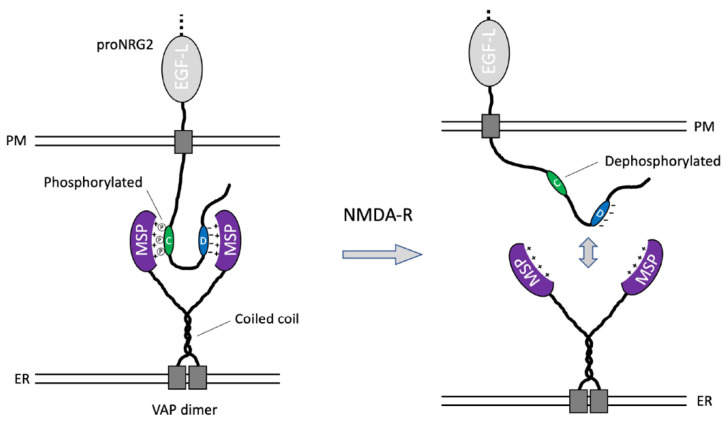
Working model of proNRG2/VAP interactions. The schematic depicts a single proNRG2 molecule cooperatively engaging with a VAP dimer via its two noncanonical, low-affinity FFAT motifs located in the C- and D-boxes. Interactions between the VAP MSP domain and the cryptic FFAT site in the C-box require phosphorylation of Ser/Thr residues. Conversely, their dephosphorylation downstream of NMDA receptor activation promotes the dissociation of proNRG2 from VAP. See Discussion for further details.

Canonical VAP interactions involve an electropositive region in its MSP domain that binds FFAT motifs in target proteins whose core conforms to the consensus sequence EFFDAxE [51]. However, recent studies have shown that experimentally validated FFAT motifs can tolerate significant sequence variations, including the lack of one or both phenylalanines and the substitution of acidic residues for phosphorylated Ser/Thr residues [51,61]. With its single phenylalanine in position 615, followed by an acidic tract (…^614^P**F**RIP**EDDE**Y**E**^624^…), the D-box likely functions as a suboptimal FFAT-like motif. By contrast, several lines of evidence from this work and our previous work [26] strongly suggest that the C-box harbors a phosphorylation-dependent VAP binding site, including: (1) bioinformatics analyses revealed five conserved potential Ser/Thr phosphorylation sites; (2) alanine substitution of these residues in proNRG2_5A dramatically increases proNRG2 electrophoretic mobility in SDS-PAGE (Figure 5B); such electrophoretic mobility shifts have also been observed for Kv2.1 [62,63] and are indicative of respectively hypo- and hyper-phosphorylated states [54]; (3) rendering these residues nonphosphorylatable has the same deleterious effect on proNRG2-VAP interactions as deleting the entire C-box (cp. Figure 4B/Figure 5B); and (4) inhibition of the Ser/Thr phosphatases PP1/PP2a and PP2b/calcineurin blocks this electrophoretic mobility shift in response to NMDA receptor activation [26]. While the exact phosphorylation sites are not known, based on the large electrophoretic mobility shift (~15 kD), it is reasonable to assume that multiple Ser/Thr residues are phosphorylated in clustered proNRG2. Furthermore, hyperphosphorylation is more likely to functionally substitute for the lack of the negative charge required for VAP binding and clustering, as previously shown for the Kv2.1 PRC motif [46,47,48,64]. While both the presence of multiple FFAT sites in a single VAP-binding protein and the existence of cryptic, phosphorylation-dependent FFAT sites have been reported previously (for reviews, see [51,65]), to our knowledge, proNRG2 is the first protein known to achieve high-affinity and activity-dependent VAP binding via a low-affinity FFAT-like motif (D-box) and a phospho-FFAT-like motif (C-box). Taking into consideration that VAP is thought to dimerize via its coiled-coil domain [50,66], we propose that these two binding sites in proNRG2 interact with two MSP domains present in a VAP dimer (Figure 6).

Our data also indicate that proNRG2 binds VAPA/B isoforms with similar affinities. In the adult mouse brain, VAPA expression predominates in the neocortex and hippocampus, while VAPB predominates in selective neuron types including α-motoneurons, where NRG1 clusters at C-bouton ER-PM junctions [22,25,48,67,68]. Whether isoform-selective VAP–proNRG2 interactions are functionally distinct is an interesting question and should be addressed in future studies.

### 3.3. Potential Functional Implications

The results of the present study, taken together with earlier studies on the cell biology and physiology of Kv2.1 and the NRG/ErbB4 signaling pathway in cortical neurons (see below), strongly suggest that proNRG2 and Kv2.1 are bifunctional proteins. The VAP-dependent formation of ER-PM junctions can be viewed as a structural function that allows both proteins to self-organize into dense surface clusters comprised of their inactive states (signaling-incompetent proform in the case of NRG2 and nonconducting potassium channel in the case of Kv2.1; [8,33,69,70,71]). These proNRG2/Kv2.1 co-clusters are concentrated on cell bodies and proximal dendrites, where they provide a spatially restricted reservoir that is optimally positioned to regulate neuron firing in response to the activation of NMDA receptors and their downstream targets. For Kv2.1, activation manifests as a transition from a nonconducting state to a conducting state [70] and as a hyperpolarizing shift in its activation kinetics [62] resulting in reduced neuron firing [72]. For NRG2, the conversion from the inactive proform to the signaling-competent ErbB4 receptor ligand involves its ectodomain shedding by ADAM10 [26]. ErbB4 signaling then negatively feeds back on NMDA receptor function [8,9] and reduces action potential firing via the downregulation of voltage-gated sodium channel currents [7]. Importantly, unlike Kv2.1, which is broadly expressed throughout the CNS [71,73,74,75], cortical NRG2/ErbB4 signaling is restricted to GABAergic interneurons [8,76]. As (patho-)physiologically relevant conditions that trigger Kv2.1/proNRG2 activation include periods of high-frequency synaptic transmission [56] and elevated extracellular glutamate levels, including those caused by hypoxia or disrupted glutamate reuptake [8,77,78,79], it is tempting to speculate that convergent NMDA receptor-dependent activation of Kv2.1-mediated currents and NRG/ErbB4 signaling endows GABAergic interneurons with particularly powerful neuroprotective mechanisms during periods of elevated excitatory activity or ischemic conditions. This would be especially relevant for fast-spiking parvalbumin-positive interneurons, virtually all of which express ErbB4 [80,81,82], due to their high metabolic demand and vulnerability to oxidative stress conditions [83,84,85]. It will be interesting in future studies to develop genetic tools to target both NRG2/ErbB4 signaling and Kv2.1 currents selectively in (fast-spiking) GABAergic interneurons and the in vivo experimental approaches to directly test their functional partnership, and to explore whether a similar partnership exists between NRG1 and Kv2.1 in the homeostatic regulation of α-motorneuron excitability [49].

## 4. Materials and Methods

### 4.1. Animals

Sprague Dawley rat (Taconic Biosciences, Germantown, NY, USA) E18 or E19 fetuses were used to prepare dissociated hippocampal neurons. Adult wild-type C57Bl6/J mice of either sex (bred in-house) were used to prepare whole brain protein extracts. Animals were treated in accordance with the NIH Animal Welfare guidelines. All procedures were approved by the NICHD Animal Care and User Committee (Animal Study Proposal: 21-074, date of approval: 4/1/2021).

### 4.2. Antibodies

See Table S1.

### 4.3. Drugs and Biochemicals

The Complete^®^ protease inhibitor cocktail and phosphatase inhibitor cocktail sets I and II were from MilliporeSigma (Burlington, MA, USA). NMDA and D-AP5 were from Tocris. NRG2-derived peptides used in GST pulldown competition assays were from Genscript. Synthetic double-stranded DNA fragments used to make various DNA constructs were from Integrated DNA technologies. Biochemicals used in IP experiments, GST pulldowns, and Western blotting were from Thermo Fisher (Waltham, MA, USA), Millipore Sigma, or Bio-Rad (Hercules, CA, USA).

### 4.4. Constructs

All proNRG2 constructs were based on the human cDNA in Gateway^®^ entry vector pENTR223.1 (accession number: BC166615.1). Unless otherwise indicated, all variants included the sequence encoding the V5 epitope tag immediately upstream of the sequence encoding the EGF-like domain. For live-labeling of proNRG2 in transfected HeLa cells, an ALFA epitope tag was used instead of V5 [86]. C- and D-box deletions and point mutations were made by inserting synthetic DNA fragments (“gBlocks”; Integrated DNA Technologies) harboring the corresponding mutations between the KpnI-AatII sites of human NRG2 in pENTR223.1. Open reading frames were transferred by Gateway^®^ recombination to the destination vectors pAAV_hSynI for AAV production and neuron-selective expression from the human Synapsin I promoter and to pcDNA-DEST40 (wild-type CMV promoter) or pDESTDV3 (attenuated CMV promoter; [22]) for transfection and expression in, respectively, HeLa and 293 cells.

Wild-type and mutant (K87D/M89D) rat VAP-A-GFP constructs in pEGFP-N1 were obtained from Addgene (Cat-#: 18,874, 18,875; [52]). Their open reading frames were amplified by PCR and cloned into the Gateway entry vector pENTR/D (Thermo Fisher), from which they were transferred to the Gateway destination vectors pAAV_hSynI for AAV production and pcDNA-DEST40 for transient HeLa cell transfections. Rat VAP-A and VAP-B isoforms, fused carboxyl-terminally to V5, were made as synthetic DNA fragments, cloned into the Gateway^®^ entry vector pENTR/D, and transferred to the pDESTDV3 or pcDNA-DEST40 destination vectors by Gateway^®^ recombination.

The GST-NRG2_CD bait construct was made by inserting a synthetic DNA fragment corresponding to amino acids 453–642 of human NRG2 (GenBank accession number NP_004874.1), encompassing the C- and D-boxes plus 30 amino acids of upstream and 16 amino acids of downstream flanking sequence, between the BamHI-EcoRI sites of pGEX-2T. Codons for the five potential phosphorylation sites Ser494, Ser497, Ser502, Thr505, and Ser511 in the C-box were replaced by Asp to ensure VAP binding in the absence of eukaryotic kinases capable of phosphorylating these sites. The empty pGEX-2T plasmid was used to express unfused GST that served as a negative control bait protein.

See below for the generation of the Kv2.1 shRNA-mediated knockdown and control vectors. All constructs made or altered in-house were verified by Sanger sequencing.

### 4.5. Primary Hippocampal Neuron Cultures

Dissociated hippocampal neurons were prepared from E19 Sprague Dawley rat pups and propagated in defined Neurobasal/B27 medium (Thermo Fisher). Neurons were seeded in 24-well or 6-well plates, either directly on poly-D-lysine-coated plastic for immunoprecipitation and Western blotting (120,000 cells/mL) or immunogold electron microscopy (200,000 cell/mL), or on poly-D-lysine-coated 12 mm #1.5 round coverslips for immunofluorescence cytochemistry (80,000 cells/mL). Cultures were maintained in Neurobasal/B27 medium, and half of the medium was changed once every week. Neurons were typically transduced between days in vitro (DIV) 10 and 17 and analyzed 10–14 days later. All experiments were performed at least 2 days following a medium change to minimize potential NMDA receptor-mediated effects on proNRG2 subcellular distribution and ectodomain shedding (see [26]).

### 4.6. 293 Cell Culture and Transfection

293 cells were used to generate protein lysates for immunoprecipitation experiments. The 293-LTV subclone was used because of its improved attachment to cell culture plastic (Cell Biolabs). The cells were maintained in DMEM supplemented with 10% fetal calf serum and grown in T25 flasks. Cultures were split 1:5–1:10 every 2–3 days. For transfections, the cells were seeded directly on plastic in 12- or 6-well plates and transfected at 60–80% density with 1 µg (12-well) or 2 µg (6-well) of plasmid DNA using the LipoD293^™^ transfection reagent (SignaGen, Frederick, MD, USA). Cells were processed for immunoprecipitation and Western blotting 24–48 h later.

### 4.7. HeLa Cell Culture and Transfection

The HeLa cell line was used to analyze proNRG2/VAP colocalization by immunocytochemistry. Cells were maintained in DMEM supplemented with 10% fetal calf serum and grown in T25 flasks. The cultures were split 1:5–1:10 every 2–4 days. For transfections, the cells were seeded on 12 mm #1.5 round coverslips in 12-well plates and transfected at 60–80% density with 0.75 µg of plasmid DNA using the GenJet™ transfection reagent (SignaGen). Cells were processed for immunocytochemistry 24 h later.

### 4.8. AAV Preparation

AAV-293 cells (Agilent, Santa Clara, CA, USA) grown in DMEM medium with 10% FBS were used for AAV production (serotype 1). Cells were seeded in 15 cm round dishes, and the medium was changed 2 h before transfection. Cells were triple-transfected at 50–60% confluence with pHelper (Agilent), pAAV-RC1 (Cell Biolabs, San Diego, CA, USA), and transfer vectors harboring transcription units for proNRG2/VAP variants or Kv2.1/NTC shRNAs. Per dish, ~15 μg of DNA at equimolar ratios (6.6 μg pHelper, 4.4 μg pRC1, and 4.4 μg transfer vector) was diluted in 500 μL of DMEM medium (Thermo Fisher) and mixed with 45 µL of LipoD293 diluted in 500 µL of DMEM. The DNA:lipid mixture was incubated for 10 min at RT (room temperature) and then added dropwise to the cells. Cultures were fed 48 h later by adding 10 mL of growth medium. A total of 72 h after the transfection, AAVs were purified from cell pellets or from conditioned supernatants. To extract the virus from the cellular fraction, cells were scraped into gradient buffer (150 mM NaCl, 10 mM MgCl_2_, 10 mM Tris-Cl pH 7.6) and lysed by multiple freeze–thaw cycles, aided by repeated passages through a 23-gauge needle. The lysates were then incubated with benzonase (MilliporeSigma) for 1 h at 37 °C and cleared by centrifugation (15 min at 4000× *g*, 4 °C). The supernatants were layered on top of an iodixanol step gradient (15, 25, 40, and 58% (*v*/*v*) in gradient buffer; MilliporeSigma), and the remaining volume was filled with gradient buffer. Ultracentrifugation was performed at 48,000 rpm using a type 70Ti fixed angle rotor (Beckman, Brea, CA, USA) for 2 h at 18 °C. Virus particles were collected from the 40% iodixanol layer and stored at 4 °C for immediate use or at −80 °C for long-term storage. AAV purification from conditioned culture supernatants was carried out using the AAV concentrator kit (Takara, Bio USA, San Jose, CA, USA). All AAV preparations were titrated in cultured hippocampal neurons and used at dilutions that yielded a widespread expression at moderate levels that were suitable for detection by Western blotting (typically 2–4 µL per 6-well or 35 mm dish) or low-to-moderate expression for immunocytochemistry and confocal microscopy (typically 0.1 −0.5 µL per 24-well), unless otherwise indicated.

### 4.9. shRNA-Mediated Kv2.1 Knockdown

Potential target sequences for the shRNA-mediated knockdown of rat Kv2.1 were identified using the Block-iT RNAi online designer tool from Invitrogen/Thermo Fisher. Four high-scoring shRNA sequences were selected and cloned as double-stranded oligonucleotides between the EcoRI-BamHI sites of the AAV vector pZacf (modified to co-express EGFP from the human Synapsin I promoter). shRNAs were tested by the immunocytochemistry and Western blotting of AAV-transduced cultured hippocampal neurons, resulting in the identification of highly effective Kv2.1 shRNA 2956 (target sequence: 5′- GGGTGTCGGTCTCCTGTTA-3′; the number indicates the position of the first nucleotide relative to the rat Kv2.1 sequence in GenBank accession number X16476.1). The nontargeting control vector contained a sequence derived from the *Photinus pyralis* luciferase gene.

### 4.10. Immunocytochemistry

For post-fixation labeling, dissociated hippocampal neurons on 12 mm #1.5 round coverslips were fixed at DIV17-20 with 4% paraformaldehyde (PFA) in PBS supplemented with 4% sucrose for 15 min at RT. Coverslips were washed extensively with phosphate-buffered saline (PBS) and then blocked and permeabilized for 30–60 min at RT in PBS containing 10% normal donkey serum (NDS) and 0.1% Triton X-100. Primary antibodies were diluted in 2% NDS/0.1% Triton X-100 in PBS, and cells were incubated in antibody solution for 1 h at RT. For proNRG2 surface labeling experiments, neurons were first blocked in detergent-free PBS containing 10% NDS and then incubated with anti-V5 in 2% NDS in PBS for 1 h at RT. Neurons were then permeabilized for 30–60 min at RT in PBS containing 0.1% Triton X-100 and 10% NDS, followed by incubation with anti-Kv2.1 and anti-ErbB4 antibodies in 2% NDS/0.1% Triton X-100 in PBS for 1 h at RT. Coverslips were washed with PBS and then incubated for 1 h at RT with fluorophore-conjugated secondary antibodies in 2% NDS/0.1% Triton X-100 in PBS. Cells were washed again with PBS, dipped in water, and mounted onto glass slides using the Mowiol/DABCO mounting medium. For experiments shown in Figure 1E,F which employ two mouse monoclonal IgG_1_ antibodies against NRG2 (clone 8D11) and Kv2.1 (clone K89/34), Kv2.1 was detected using a Zenon conjugate, following the manufacturer’s instructions (Invitrogen, Waltham, MA, USA). All antibody solutions were cleared prior to use by centrifugation at 10,000× *g* for 10 min at 4 °C.

For immunocytochemistry of proNRG2/VAP in the HeLa cell line, cells were plated onto 12 mm #1.5 round coverslips in 12-well plates and transfected the next day with ALFA-tagged proNRG2 and GFP- or V5-tagged VAP constructs. The BACE/metalloproteinase inhibitors LY2886721 (1 µM) and GM6001 (10 µM) were added to prevent ectodomain shedding. The next day, surface proNRG2 was live-labeled with ALFA-tag nanobody (1:200; Synaptic Systems, Göttingen, Germany) for 30 min at 37 °C/5% CO_2_. Cells were then washed with prewarmed HeLa medium containing the shedding inhibitors LY2886721 and GM6001, fixed with 4% PFA in PBS, blocked, and permeabilized as described above. To visualize V5-tagged VAP-A/B, cells were additionally incubated with anti-V5 in 2% NDS/0.1% TX-100 in PBS for 1 h at RT. Subsequent washes, secondary antibody incubations, and mounting were carried out as described above. DAPI was included in all experiments to label nuclei.

### 4.11. Confocal Microscopy

All images were acquired under minimally saturating conditions, unless otherwise noted. LSM780 and LSM710 confocal microscopes (Zeiss, Jena, Germany) were used to collect image stacks of neuron cell bodies and proximal dendrites using a 63× oil objective and 2× digital zoom. Unless indicated otherwise, laser intensities and digital gain for quantitative analyses were set using signals from a representative control neuron, and then the same settings were used for the analyses of all other experimental conditions (Kv2.1-KD, proNRG2 variants, etc.). The image stacks were then projected in Z using the maximum intensity method. For the images collected in Figure 1A, laser intensity and digital gain were set for each individual coverslip and used for all images collected from that coverslip. The Z-projected superresolution images of proNRG2 puncta under Kv2.1 knockdown conditions shown in Figure 1G were acquired on an Airyscan LSM880 (Zeiss), with the laser intensity and digital gain for proNRG2 and Kv2.1 set using a representative neuron from a control coverslip. The representative images of proNRG2/VAP-transfected HeLa cells shown in Figure 3B,D were acquired as single planes, and laser settings were optimized for each image.

### 4.12. Immunogold Electron Microscopy

Dissociated hippocampal neurons were plated at a density of 200,000 cells per mL in 6-well culture dishes coated with poly-D-lysine and transduced at DIV10 with an AAV for proNRG2 under the control of the human synapsin I promoter. Thirteen days post-transduction, neurons were live-labeled for 30 min with mouse monoclonal anti-NRG2 antibody 8D11 in conditioned culture medium (diluted 1:25 from hybridoma supernatant) at 37 °C/5% CO_2_. Unbound antibody was washed off using temperature- and CO_2_-equilibrated conditioned supernatant collected from age- and density-matched untransduced hippocampal neurons (two brief rinses and one 5 min wash). The neurons were then incubated with gold-conjugated anti-mouse secondary antibody (Nanogold, Nanoprobes, Yaphank, NY, USA) in conditioned neuron medium for 30 min and washed as described above. Notably, all antibody incubation and wash steps were performed in the presence of 50 µM of AP5 to block NMDA receptors. Neurons were then fixed with 2% (*v*/*v*) glutaraldehyde (Electron Microscopy Sciences, Hatfield, PA, USA) in PBS for 30 min at RT and then overnight at 4 °C. The nanogold label was silver-enhanced (HQ kit, Nanoprobes), treated with 0.2% osmium tetroxide in 0.1 M phosphate buffer at pH 7.4 for 30 min, *en bloc* stained with 0.25% uranyl acetate in acetate buffer at pH 5.0 for 1 h, dehydrated in a series of graded ethanols, and embedded in epoxy resin [87]. Thin sections were examined on a JEOL 1200 EX transmission electron microscope, and images were collected with a digital CCD camera (AMT XR-100, Advanced Microscopy Techniques, Woburn, MA, USA).

### 4.13. Image Analysis

For all quantitative analyses, images were acquired with minimal pixel saturation. Within the same experiment, laser intensities and gains were kept constant across different experimental conditions. Upon acquisition, image stacks of cell bodies and proximal dendrites were projected in Z using maximum intensity projection in Zen (Zeiss). Correlation analysis of proNRG2/Kv2.1 cluster sizes on ErbB4+ neurons (Figure 1C) was conducted using ImageJ/FIJI. ROIs were defined as described above, and clusters were identified using the “analyze particles” feature, setting a lower pixel value threshold of 40 and a minimal particle size of 0.05 µm^2^ for both proNRG2 and Kv2.1. Size and number of proNRG2 puncta on ErbB4+ neurons following Kv2.1 knockdown by shRNA (Figure S2) were analyzed using ImageJ/FIJI. Areas including the cell body and proximal dendrites were defined as regions of interest (ROIs) and analyzed using the “analyze particles” feature with a lower threshold of 51 and an upper threshold of 255. Spatial relationship between the proNRG2 C/D-box deletion variants and Kv2.1 (Figure 4E) was analyzed using the plot profile feature in ImageJ/FIJI. Analysis of NRG2 cluster size in Figure 4F was done as described for Figure 1C, but with a lower threshold of 75.

### 4.14. Immunoprecipitation

For cultured cells, 6-well plates were placed on ice, and wells were washed twice with cold PBS. Cells were lysed by adding 600 µL of IP buffer per well. The IP buffer composition was as follows: 300 mM NaCl, 50 mM Tris-Cl pH 7.5, 1% Triton X-100, and Complete^®^ protease inhibitor cocktail and phosphatase inhibitor cocktails (Sets I and II; all inhibitors from MilliporeSigma). After a 5 min incubation on ice, lysates were cleared by centrifugation (10 min at 2300× *g*, 4 °C), and aliquots of the supernatants were set aside to prepare whole-cell lysate samples for SDS-PAGE/Western blotting. IP buffer-equilibrated Protein A magnetic beads (25 µL per reaction; Thermo Fisher) and IP antibodies (2 µg) were then added to the remainder of the cleared cell lysates (500 µL). The IPs were incubated with head-over-tail rotation for 2 h at 4 °C. Following three washes with 750 µL of IP buffer, the bound proteins were eluted and made into SDS-PAGE samples by adding 100 µL of LDS sample buffer (Thermo Fisher) and incubating them for 10 min at 80 °C.

### 4.15. GST Pulldown Assays

Expression of GST and GST-NRG2_CD bait proteins in the *E. Coli* host strain BL21(DE3) (Thermo Fisher) was induced with 0.4 mM IPTG (Isopropyl ß-D-1-thiogalactopyranoside) for 4 h at 37 °C with shaking. Cells were harvested by centrifugation and homogenized by sonication in cold PBS (250 µL per 2 mL of cell suspension). Triton X-100 was added to a final concentration of 0.1%, and homogenates were incubated for 5 min on ice. Homogenates were then cleared by centrifugation for 10 min at 2300× *g* and 4 °C. Supernatants were then diluted to 1 mL with cold binding buffer (PBS + 0.1% Triton X-100) and incubated with 75 µL of Glutathione-conjugated magnetic beads (Thermo Fisher) equilibrated in binding buffer for 2 h at 4 °C with head-over-tail rotation. Following three wash steps with binding buffer and two wash steps with IP buffer (see above), bait protein-loaded magnetic beads were split into individual pulldown reactions and pre-incubated with competing C- and D-box peptides (20 µm final concentration) or vehicle (water) for 2 h at 4 °C with head-over-tail rotation, followed by three washes with IP buffer. Lysates from untransduced neurons in 6-well plates were prepared as described above (see Section 4.14) and used to pull down VAP in the presence of competing peptides (20 µM) or vehicle for 2 h at 4 °C with head-over-tail rotation. Following three washes with 750 µL of IP buffer, bound proteins were eluted and made into SDS-PAGE samples by adding 100 µL of LDS sample buffer and incubating them for 10 min at 80 °C.

### 4.16. Western Blotting

Samples for SDS-PAGE were generated by mixing protein lysates or IP fractions with 4× LDS buffer (Thermo Fisher) and β-Mercaptoethanol (5% final concentration; MilliporeSigma), followed by heating at 80 °C for 10 min. Proteins were size-fractionated on 4–15% Mini-Protean TGX precast gels (Bio-Rad) and electroblotted onto nitrocellulose using semi-dry transfer (Trans-blot turbo transfer system; Bio-Rad). Membranes were blocked with 3% BSA (MilliporeSigma) in Tris-buffered saline containing 0.1% Tween-20 (TBS/T; Pierce) and incubated with primary antibody in the blocking solution for 1–2 h at RT or overnight at 4 °C. After several washes with TBS/T, membranes were incubated with horseradish peroxidase (HRP)-conjugated secondary antibodies (Jackson Immunoresearch, West Grove, PA, USA) for 1 h at RT. Signals were detected by chemiluminescence (Super Signal West Pico PLUS; Thermo Fisher Scientific) using a ChemiDoc MP imager (Bio-Rad). Rhodamine-conjugated anti-tubulin or anti-GAPDH antibodies were used as loading controls (Bio-Rad) and detected in fluorescent mode. Densitometry analyses were performed using Image Lab software (Bio-Rad).

### 4.17. Statistics

Statistical analyses were performed in Prism9 (GraphPad, San Diego, CA, USA). One-way ANOVA with Dunnett’s multiple comparison test was used to analyze the relationship between AAV transduction volume and proNRG2 cluster size (Figure 1B), and the effects of competing peptides on VAP signals in the GST pulldown experiments (Figure 5F). A Pearson coefficient was calculated to correlate proNRG2 and Kv2.1 cluster sizes, as shown in Figure 1C. A nonparametric Kruskal–Wallis test with Dunn’s multiple comparisons was used to analyze the effect of Kv2.1 knockdown on the number of ErbB4+ GABAergic interneurons with proNRG2 (Figure 1H). Welch’s *t* test was used to compare the mean proNRG2 puncta number per neuron and the mean puncta size between neurons transduced with Kv2.1 shRNA and neurons transduced with nontargeting control shRNA (Figure S2). One-way ANOVA with Tukey’s multiple comparison test was used to analyze the effects of C- and D-box deletions on the proNRG2 cluster size (Figure 4F). A Kruskal–Wallis test with Dunn’s multiple comparisons was used to test the effect of NMDA receptor stimulation on VAP co-immunoprecipitation, as shown in Figure 5H.

## Data Availability

Data not contained within this article or Supplementary Materials is available upon request.

## Supplementary Materials

The following supporting information can be downloaded at: https://www.mdpi.com/article/10.3390/ijms24032908/s1, Figure S1: ProNRG2 clusters at bona fide ER-PM junctions; Figure S2: Effect of Kv2.1 knockdown on proNRG2 cluster size and number; Figure S3; Widespread subcellular distribution of proNRG2ΔCΔD; Table S1: Antibodies used in this study.
