# Supplementary material for: ER-PM Junctions on GABAergic Interneurons Are Organized by Neuregulin 2/VAP Interactions and Regulated by NMDA Receptors
Source: Int J Mol Sci. 2023 Feb 2;24(3):2908. doi: 10.3390/ijms24032908 (PMC9917868; doi:10.3390/ijms24032908)
Supplement: Supplementary file 1 [file ijms-24-02908-s001.zip › ijms-2172403-supplementary.pdf]

Supplementary Materials

Contents:

Figure S1 (related to Figure 1): ProNRG2 clusters at bona fide ER-PM junctions.

Figure S2 (related to Figure 1): Effect of Kv2.1 knockdown on proNRG2 cluster size and number.

Figure S3 (related to Figure 4): Widespread subcellular distribution of proNRG2ΔCΔD.

Supplementary Table S1: Antibodies used in this study

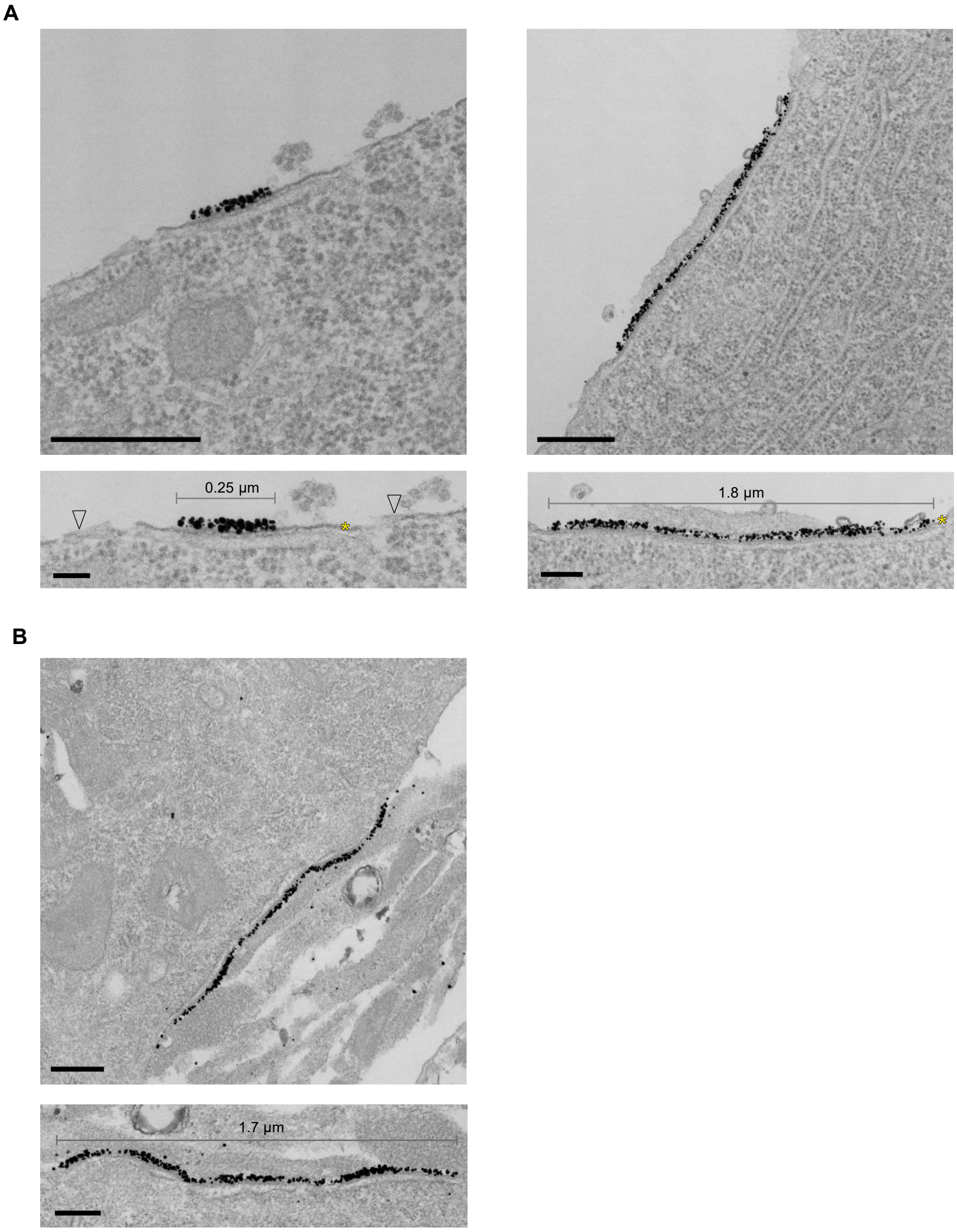

Figure S1 (related to Figure 1): ***A***, Low-magnification immunogold EM images (*top*) and unshaded versions of the magnified ER-PM junctions (*bottom*) shown in **Fig. 1*D***. ***B***, Low-magnification immunogold EM image (*top*) and unshaded version of the magnified ER-PM junction (*bottom*) shown in **Fig. 1*J***. Scale bars: A,B (overviews) = 500 nm; A, (magnified) = 100 nm (left) and 200 nm (right); B (magnified) = 200 nm (right).

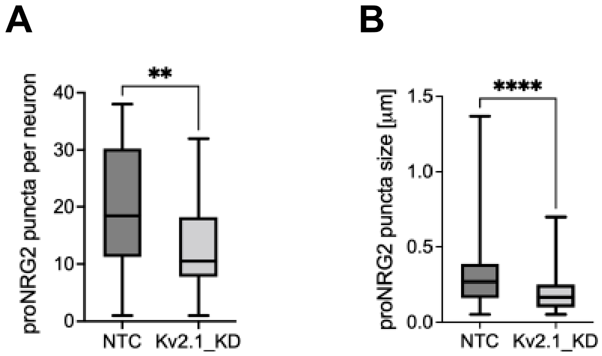

Figure S2 (related to Figure 1): Effects of Kv2.1 knockdown on NRG2 puncta number and size. ***A***, Kv2.1 knockdown moderately reduces the mean number of proNRG2 puncta per neuron (NTC: 19.7±2.0 vs. Kv2.1-KD: 11.3±1.4). n= 32 (*NTC*) and 30 (*Kv2.1-KD*) neurons from 3 independent experiments. **, $p<0.01$ (unpaired t test). ***B***, Kv2.1 knockdown also reduces the mean size of endogenous proNRG2 puncta (*NTC*: 0.30±0.007 μm^2 vs. *Kv2.1-KD*: 0.19±0.12 μm^2). n= 631 (*NTC*) and 386 (*Kv2.1-KD*) puncta from 3 independent experiments. ****, $p<0.0001$ (Welch's test).

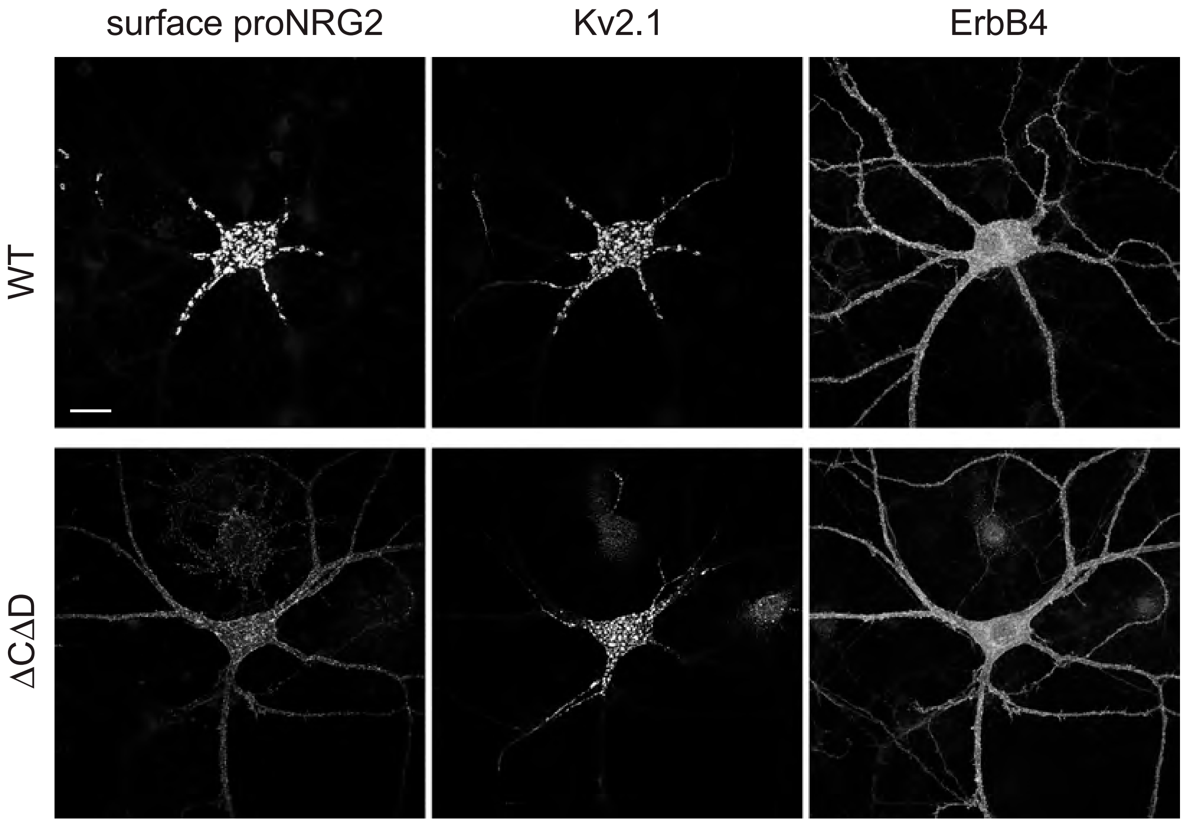

Figure S3 (related to Figures 1 and 4): Representative low-magnification micrographs of ErbB4+ GABAergic interneurons transduced with WT proNRG2 (*top*) or proNRG2ΔCΔD (*bottom*), and surface-labeled for proNRG2 using anti-V5 (*left*). Unlike WT proNRG2 that, like Kv2.1 (*center*), forms large clusters on cell bodies and proximal dendrites, proNRG2ΔCΔD is broadly and evenly distributed throughout the entire neuron including in distal dendrites. Note that the amount of proNRG2ΔCΔD AAV used here was approximately fourfold higher than in **Fig. 4*D*** to facilitate detection. Scale bar = 25 μm.

Supplementary Table S1: Antibodies used in this study

Target	Species / clonality	Clone	Supplier	Catalog / Reference	RRID
NRG2	Mouse monoclonal	8D11	Millipore/Sigma	MABN1853	Not available
	Rabbit polyclonal	N/A	Millipore/Sigma	ABN1654	
V5	Mouse monoclonal	SV-Pk1	Bio-Rad	MCA-1360	AB_322378
FluoTag-X2 anti-ALFA Sulfo Cy3	Camelid single-domain antibody	1G5	NanoTag Biotechnologies	N1502-SC3-L	Not available
VAPA/B	Mouse monoclonal	N479/107	Antibodies Incorporated	75-496	AB_2756370
VAPA	Rabbit polyclonal	N/A	Proteintech	15275-1-AP	AB_2256991
VAPB	Rabbit polyclonal	N/A	Proteintech	14477-1-AP	AB_2288297
Kv2.1	Mouse monoclonal	K89/34	Antibodies Incorporated	75-014	AB_2877280
ErbB4	Rabbit polyclonal		Custom	Vullhorst et al., 2009	Not available
GFP	Mouse monoclonal	N86/6	Antibodies Incorporated	75-131	AB_10671445
Tubulin	Human FAB, recombinant[1]		Bio-Rad	12004165	AB_2884950
GAPDH	Human FAB, recombinant[1]		Bio-Rad	12004167	AB_2884950

Notes:

[1] Directly conjugated to rhodamine; used in Western blotting to control for equal sample loading.
